# Renoprotective Effects of Solid-State Cultivated *Antrodia cinnamomea* in Juvenile Rats with Chronic Kidney Disease

**DOI:** 10.3390/nu15214626

**Published:** 2023-10-31

**Authors:** You-Lin Tain, Guo-Ping Chang-Chien, Sufan Lin, Chih-Yao Hou, Chien-Ning Hsu

**Affiliations:** 1 Department of Pediatrics, Kaohsiung Chang Gung Memorial Hospital, Kaohsiung 833, Taiwan; tainyl@cgmh.org.tw; 2 College of Medicine, Chang Gung University, Taoyuan 330, Taiwan; 3 Institute for Translational Research in Biomedicine, Kaohsiung Chang Gung Memorial Hospital, Kaohsiung 833, Taiwan; 4 Institute of Environmental Toxin and Emerging-Contaminant, Cheng Shiu University, Kaohsiung 833, Taiwan; guoping@csu.edu.tw (G.-P.C.-C.); linsufan2003@csu.edu.tw (S.L.); 5 Super Micro Mass Research and Technology Center, Cheng Shiu University, Kaohsiung 833, Taiwan; 6 Center for Environmental Toxin and Emerging-Contaminant Research, Cheng Shiu University, Kaohsiung 833, Taiwan; 7 Department of Seafood Science, National Kaohsiung University of Science and Technology, Kaohsiung 811, Taiwan; chihyaohou@webmail.nkmu.edu.tw; 8 Department of Pharmacy, Kaohsiung Chang Gung Memorial Hospital, Kaohsiung 833, Taiwan; 9 School of Pharmacy, Kaohsiung Medical University, Kaohsiung 807, Taiwan

**Keywords:** *Antrodia cinnamomea*, chronic kidney disease, children, gut microbiota, short-chain fatty acid, hypertension, renin-angiotensin system, trimethylamine-N-oxide

## Abstract

*Antrodia cinnamomea* (AC), a medicinal mushroom, has multiple beneficial actions, such as acting as a prebiotic. The incidence of chronic kidney disease (CKD) in children has steadily increased year by year, and CKD is related to gut microbiota dysbiosis. Herein, we investigated the renoprotection of solid-state cultivated AC in adenine-induced CKD juvenile rats. CKD was induced in 3-week-old male rats by feeding with adenine (0.5%) for three weeks. Treated groups received oral administration of AC extracts at either a low (10 mg/kg/day) or high dose (100 mg/kg/day) for six weeks. At nine weeks of age, the rats were sacrificed. Renal outcomes, blood pressure, and gut microbiome composition were examined. Our results revealed that AC treatment, either low- or high-dose, improved kidney function, proteinuria, and hypertension in CKD rats. Low-dose AC treatment increased plasma concentrations of short-chain fatty acids (SCFAs). Additionally, we observed that AC acts like a prebiotic by enriching beneficial bacteria in the gut, such as *Akkermansia* and *Turicibacter*. Moreover, the beneficial action of AC against CKD-related hypertension might also be linked to the inhibition of the renin-angiotensin system. This study brings new insights into the potential application of AC as a prebiotic dietary supplement in the prevention and treatment of pediatric CKD.

## 1. Introduction

Emerging evidence supports that aromatic and medicinal plants are nutraceuticals exerting beneficial effects on human health. In particular, medicinal mushrooms are a group of fungi that have been broadly utilized for the treatment of several human diseases [1]. *Antrodia cinnamomea* (AC), also known as *Taiwanofungus camphoratus*, is a species of fungus used as a medicinal mushroom that is endemic to Taiwan. So far, multiple compounds, such as polysaccharide, polyphenol, and triterpenoid, have been identified from AC [2,3]. Prior work revealed that the pharmacological actions of AC cover antioxidant, hepatoprotective, antihyperlipidemic, anti-inflammatory, anticancer, and immunomodulatory activities [2,3].

Chronic kidney disease (CKD) is a progressive disease that can originate in the earliest stage of life [4]. Regardless of recent advances in the treatment of CKD, there is still a rising global prevalence [5]. As a result, heightened efforts are needed to develop effective interventions for preemption, especially in childhood [6]. We previously demonstrated that adenine-fed juvenile rats displayed major features of CKD, including reduced kidney function, elevated blood pressure (BP), renal hypertrophy, and kidney injury [7].

The gut microbiome and microbial metabolites have a key role in CKD and its associated complications [8,9]. CKD has been shown to alter the gut microbiome, leading to increased tryptophan-derived uremic toxins, the dysregulation of short-chain fatty acids (SCFA) and their receptors, and increases in trimethylamine-N-oxide (TMAO) [8,9]. Prior work indicated that microbiota-targeted therapeutics could prevent pediatric CKD and related hypertension [10,11]. Increasing evidence supports the fact that medicinal mushrooms act as prebiotics and contribute to the improvement of host health [12]. However, the influence of AC on gut microbiome in pediatric CKD and hypertension is still largely unknown.

As wild fruiting bodies of *Antrodia cinnamomea* (AC) are in short supply and expensive, several artificial cultivation techniques have been established [3]. Solid-state fermentation has been established to supplement the growing demand for AC. The extract of solid-state cultures of AC exhibited an acute BP-lowering effect in spontaneously hypertensive rats via regulating the renin-angiotensin system (RAS) [13]. Another study revealed that AC could prevent the decline of kidney function in a rat CKD model [14]. This research was, hence, performed to clarify whether AC treatment can protect juvenile rats against CKD progression and high BP and explore the protective mechanisms, focusing on the gut microbiome and microbial-derived metabolites.

## 2. Materials and Methods

### 2.1. Preparation of Solid-State Cultures of Antrodia camphorata

The Antrodia camphorata was obtained from Antrodia camphorata BCRC35716 (Food Industry Research and Development Institute, Hsinchu, Taiwan). As described previously, the mycelium culture was maintained on Petri dishes containing malt extract agar media [15]. A mycelium culture on agar was inoculated into 3 L of preculture medium in a 3.5-L flask and incubated for 2 weeks at 25 °C on a rotary shaker (120 rpm). Later, the mycelia were inoculated in a 5-L fermenter for 3 months. Fungal mycelium was then harvested and freeze-dried. Supercritical fluid extraction was utilized for extracting the Antrodia camphorata fruiting body from solid-state cultures. The extract was filtered and stored at 4 °C until used.

### 2.2. Animal Model

Three-week-old male Sprague Dawley rats, obtained from BioLASCO Taiwan Co., Ltd. Taipei, Taiwan, were housed in our AAALAC-accredited animal center. The animal studies were conducted according to the Animal Research: Reporting of In Vivo Experiments (ARRIVE) guidelines and approved by the Institutional Animal Ethics Committee at our hospital (Permit # 2022081802).

Five groups of rats with a count of eight for each group were used to assess the protection of two doses of AC extracts against CKD: the control group (C), the CKD group, the control rats treated with low-dose AC group (C + LAC), the CKD rats treated with low-dose AC (CKD + LAC) group, and the CKD rats treated with high-dose AC group (CKD + HAC). We used an established a pediatric CKD model consisting of feeding 3-week-old juvenile rats with chow containing 0.5% adenine for 3 weeks [10]. Treated groups received oral administration of AC extracts at either a low (10 mg/kg BW/day) or high doses (100 mg/kg BW/day) for 6 weeks between 3 and 9 weeks of age. The dosage was chosen according to previous research on rats [13]. As males are more vulnerable to CKD and hypertension than females [16], only male rats were evaluated in the experiment.

BP was determined by the CODA tail-cuff system (Kent Scientific Corporation, Torrington, CT, USA) in rats over time at 3, 5, 7, and 9 weeks. For each rat, five measurements were recorded at each time point. Rats were sacrificed at nine weeks of age. Urine was collected in a metabolic cage (Nalgene; Nalge Nunc International, Rochester, NY, USA) for the determination of total protein and clearance of creatinine (CCr). Before sacrificing, stool samples were collected in the morning and stored at −80 °C. Blood samples were collected in tubes containing heparin. One kidney was removed, and the cortex and medulla were then dissected and snap-frozen in liquid nitrogen. The other kidney was fixed in 10% formalin and then embedded in paraffin wax. Duplicate sections from each block were cut 5-μm thick and stained with H&E and examined, blinded for grade score of glomerular and tubulointerstitial injury as described previously [17].

### 2.3. Measurement of Creatinine Concentration by HPLC

Creatinine concentration was measured by HPLC, as described previously [10]. We used an Agelient 1200 (Agilent Technologies, Wilmington, DE, USA) controller and pump to deliver 96% eluent A (10 mM ammonium phosphate buffer, pH adjusted to 7.4) and 4% eluent B (100% HPLC-grade methanol) at 1.0 mL/min, with a column temperature of 25 °C. A symmetry C18 reversed-phase column (250 × 3.00 mm i.d., 5 µm; Waters) fitted with a 10 mm × 10 mm i.d., 5 µm C18 guard column was used. Twenty-microliter samples were injected by an autosampler, and the eluted products were measured by an absorbance detector at a wavelength of 235 nm. The sample was acidified to pH 2.24 with phosphorous acid to precipitate protein. Plasma and urine samples were ultrafiltered at 13,000 rpm for 10 min, with centrifugation filters.

### 2.4. Measurement of SCFAs by GC-MS

A total of six SCFA levels in the plasma were determined, including acetic acid, butyric acid, propionic acid, isobutyric acid, valeric acid, and isovaleric acid. Samples were analyzed in duplicate by gas chromatography–mass spectroscopy (GC–MS) on a single quadruple mass analyzer [10]. A 7890B GC system coupled with an automated sampler (Agilent Technologies, Wilmington, DE, USA) was applied to carry out all analyses. The GC system was fitted with a DB-FFAP column (30 cm × 0.25 mm, 0.25 µm; Agilent Technologies). The injector inlet and transfer line temperature were set to 250 °C, with injection volume set to 1 µL and the split ratio at 5:1.

### 2.5. Measurement of TMAO by LC–MS

Plasma concentrations of dimethylamine (DMA), trimethylamine (TMA), and TMAO were measured in duplicate by liquid chromatography-MS (LC–MS) [10]. An Agilent 6410 Series Triple Quadrupole MS (Agilent Technologies) coupled with an electrospray ionization source was used to carry out all analyses. The internal standard diethylamine was used for quantification as well as to calculate recovery. DMA, TMA, and TMAO were monitored using electrospray ionization in positive-ion mode with multiple reaction monitoring of precursor and characteristic production transitions of *m*/*z* 46.1→30, *m*/*z* 60.1→44.1, and *m*/*z* 76.1→58.1, respectively.

### 2.6. 16S rRNA Gene Sequencing and Analysis

The composition of the gut microbiota was assessed by analyzing 16S rRNA gene sequences, as we described previously [10]. All samples were thawed on ice and DNA was extracted from each fecal sample. Sequencing of the full-length 16S rRNA (V1-V9 hypervariable) was carried out at the Biotools Co., Ltd. (New Taipei City, Taiwan). The full-length 16S rRNA gene was amplified with barcoded 16S gene-specific primers for multiplexed SMRTbell library (PacBio, Menlo Park, CA, USA) preparation and sequencing procedures. QIIME2 was utilized to process data from high-throughput 16S rRNA sequencing [18]. From the amplicon sequence variants (ASVs) sequences, a phylogenetic tree was created using FastTree (QIIME2). Sequencing analysis included α- and β-diversity analysis and different taxa analysis. The α-diversity indices, including Faith’s PD index and Shannon’s index were calculated. PLS-DA was implemented based on the unweighted UniFrac distance. ANOSIM was also used to assess β-diversity. LEfSe was used to determine the differentially abundant taxa with LDA > 4.

### 2.7. Analysis of Renal Gene Expression by qPCR

RNA was extracted from the renal cortical tissues. Renal gene expression of SCFA receptors and RAS components were analyzed in duplicate by quantitative polymerase chain reaction (qPCR) using SYBR Green; results were normalized to the 18S ribosomal RNA (R18S) [10]. Four SCFA receptors were analyzed, including G protein-couple receptor 41 (GPR41), GPR43, GPR109A, and olfactory receptor 78 (OLFR78). In addition, we analyzed the following RAS components, including renin, (pro)renin receptor (PRR), angiotensin-converting enzyme (ACE), and angiotensin II type 1 receptor (AT1R). These primers are shown in Table 1. The relative mRNA expression levels of genes were calculated using the comparative threshold cycle (CT) method. We calculated the fold-increase in the target gene, relative to the reference gene, using formula 2^−ΔΔCT^.

### 2.8. Statistics

Quantitative data are given as means ± the standard error of the mean (SEM). Statistical analyses were conducted with one-way ANOVA. A *p*-value < 0.05 was considered statistically significant, and Tukey’s post hoc test was applied if the *p*-value was < 0.05. Statistical analysis was carried out by SPSS Inc., Chicago, IL, USA.

## 3. Results

### 3.1. Anthropometrics, Renal Outcome, and Blood Pressure

No mortality was observed in most groups, except one control rat died right after being treated with a low dose of AC. Table 2 illustrates that adenine-treated rats in the CKD, CKD + LAC, and CKD + HAC groups had greater body weight (BW) gain compared with the C and C + LAC rats (Table 2). The kidney weight (KW) to BW ratio exhibited similar patterns. Blood creatinine (Cr) concentration was higher in the CKD and CKD + HAC groups than in the other three groups (Table 2). CKD caused a ~30% reduction in the clearance of Cr (CCr, an index of kidney function), which was restored by low- or high-dose AC treatment. In addition, proteinuria was heavier in the CKD and CKD + HAC groups compared to other groups (Table 2).

**Figure 1:**
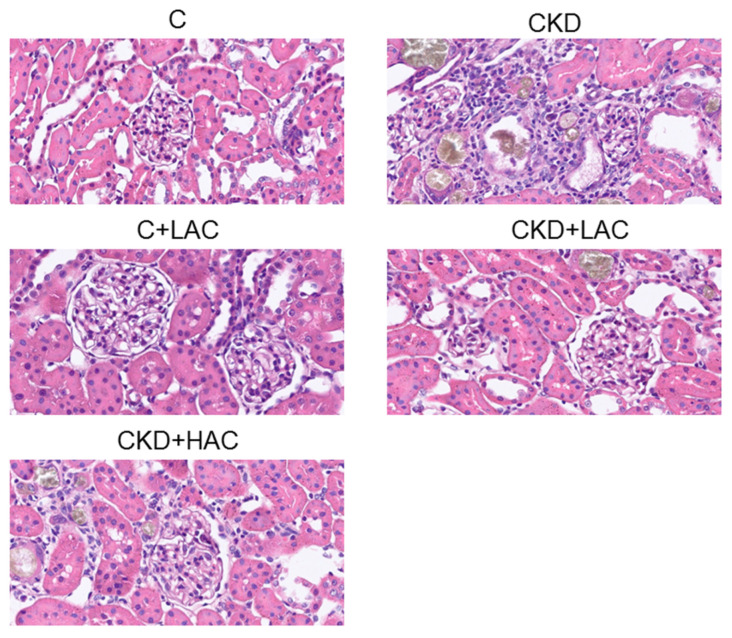
Representative hematoxylin and eosin (H&E) staining of kidney tissue from rats at 9 weeks of age.

Figure 1 illustrates the H&E histological staining of kidney tissue in rats. In line with prior research [7,19], adenine-treated rats displayed adenine crystalline deposits, thickness of glomerular and tubular basement membranes, interstitial inflammatory infiltrates, segmental necrosis, dilated tubules, and tubular atrophy on H&E staining in kidneys (Figure 1). As observed (Table 1), the adenine-induced CKD group had more severe tubulointerstitial damage and glomerular injury than the C group. The H&E histological staining of CKD + LAC and CKD + HAC groups revealed some areas of adenine crystalline deposits within kidney tubules, but the majority of glomeruli were normal (Figure 1). The grade score of glomerular injury was significantly improved by low- or high-dose AC treatment. However, the tubulointerstitial injury was attenuated but not eliminated by AC treatment.

**Figure 2:**
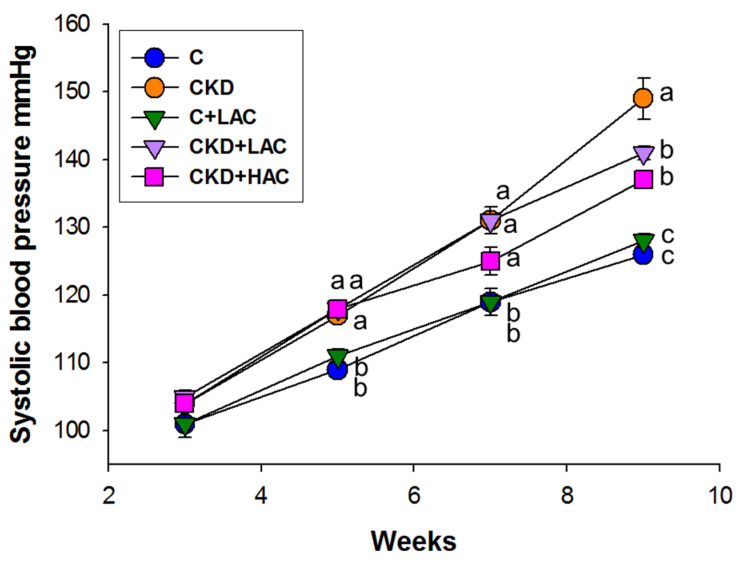
Systolic blood pressure at age 3 to 9 weeks. N = 7–8/group; statistical analysis by one-way ANOVA, *p* < 0.05. Letters represent the differences between groups.

Figure 2 illustrates that adenine-induced CKD caused an increase in systolic BP from five to nine weeks. At 9 weeks of age, the CKD group exhibited a higher systolic BP (149 ± 3 mmHg) compared with the C group (126 ± 1 mmHg, *p* < 0.05). These increases in systolic BP were partially prevented by low- or high-dose AC treatment (CKD + LAC: 141 ± 1 mmHg; CKD + HAC: 137 ± 1 mmHg).

In total, adenine feeding caused a decrease in kidney function, renal hypertrophy, proteinuria, glomerular and tubulointerstitial injury, and elevation of BP in juvenile rats, which mimic the major features of pediatric CKD. Low-dose AC treatment improved kidney function impairment, kidney damage, proteinuria, and hypertension. Similarly, a high dose was found to improve adverse renal outcomes.

### 3.2. Plasma SCFA Levels and Renal SCFA Receptors

SCFAs are major gut microbiota-derived metabolites and have an important role in modulating renal physiology and BP [20]. Table 3 reveals the impact of adenine and AC on plasma concentrations of SCFA in rats at 9 weeks of age. Low-dose AC significantly increased plasma concentrations of acetic acid, propionic acid, isobutyric acid, butyric acid, and isovaleric acid in the C + LAC group compared with the C group. By comparison, high-dose AC caused a higher plasma isobutyric acid level than the CKD group.

**Figure 3:**
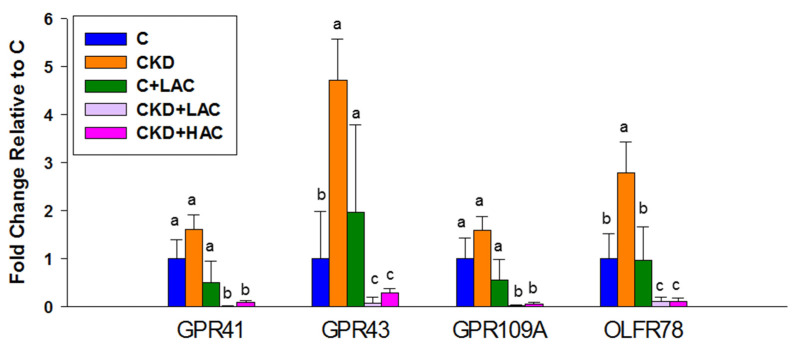
Renal gene expression of G protein-coupled receptor 41 (GPR41), GPR43, GPR109A, and olfactory receptor 78 (OFLR78) of rats at age 9 weeks. N = 7–8/group. Letters represent the differences between groups.

We then determined the renal expression of SCFA receptors, including GPR41, GPR43, GPR109A, and OLFR78. Figure 3 indicates that the CKD group has a higher expression of GPR43 and OLFR78 than the C group. Increased expression of GPR43 and OLFR78 was suppressed by low- or-high-dose AC treatment. In addition, AC treatment, either low- or high-dose, significantly reduced GPR41 and GPR109A expression compared to other groups (Figure 3).

### 3.3. DMA, TMA, and TMAO

Trimethylamine N-oxide (TMAO), a uremic toxin, is another important microbial metabolite [21]. TMAO is mainly formed from trimethylamine (TMA), and both can be converted to dimethylamine (DMA). Hence, in the current study we simultaneously measured DMA, TMA, and TMAO to offer a full scope of the TMA–TMAO metabolic pathway. As observed (Table 4), no differences in DMA, TMA, and TMAO in the plasma among the five groups were seen.

### 3.4. Gut Microbiota Composition

**Figure 4:**
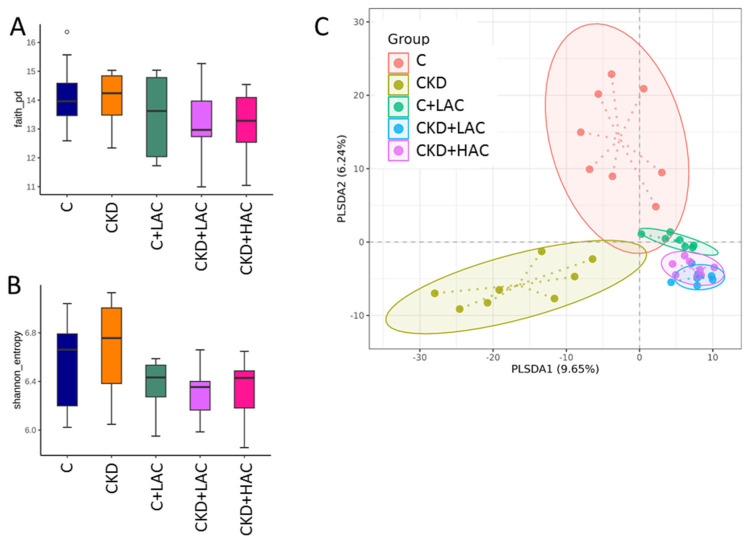
The two metrics of α-diversity: (**A**) Faith’s phylogenetic diversity (PD) index and (**B**) Shannon index are presented. (**C**) β-diversity analysis using partial least squares discriminant analysis (PLS-DA). Each dot is a sample, and the dot color represents the grouping for that sample. N = 7–8/group.

We next compared the differences in the gut microbiota among the five groups. Microbiome data can be assessed using either α-diversity (within-sample diversity) or β-diversity (between-sample diversity) metrics [22]. Figure 4 illustrates the microbial α- and β-diversity of rats at age 9 weeks. Comparison of changes in α-diversity characterized by Faith’s phylogenetic diversity (PD) index (Figure 4A) and the Shannon index (Figure 4B) showed no differences. As presented in Figure 4C, β-diversity was visualized by a partial least squares discriminant analysis (PLS-DA) plot and five distinct clusters were defined. Analysis of similarities (ANOSIM) of gut microbiota based on unweighted unifrac distance analysis showed significant differences between each group (All *p* < 0.05).

**Figure 5:**
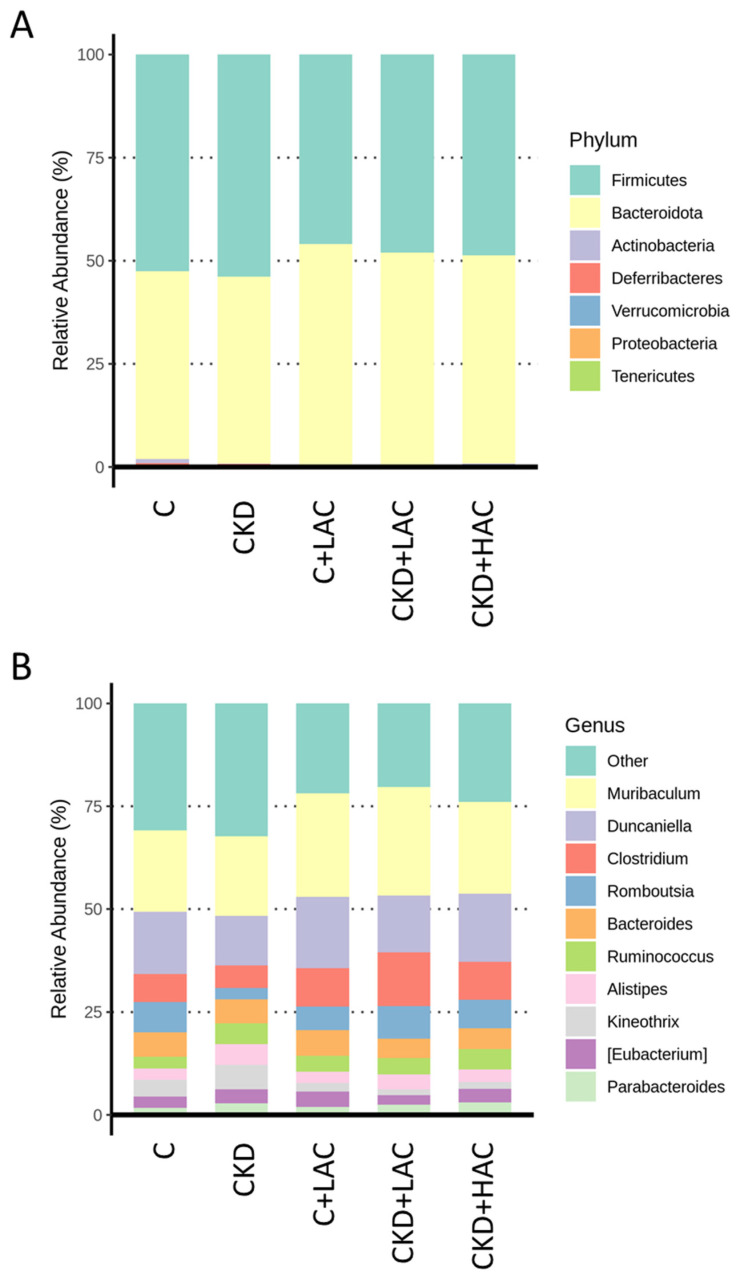
Relative abundance of dominant intestinal microflora in rats at (**A**) phylum and (**B**) genus levels.

The microbiota compositions were analyzed at phylum (Figure 5A) and genus levels (Figure 5B). The rat gut microbiota is mainly composed of two dominant phyla, *Firmicutes* and *Bacteroidota*, and by other subdominant phyla including *Actinobacteria*, *Deferribacteres*, *Verrucomicrobia*, and *Proteobacteria*. This pattern closely resembled those shown in former studies on rats [7,8,10]. Figure 5B reveals that *Muribaculum*, *Duncaniella*, *Clostridium*, *Romboutsia*, *Bacteroides*, and *Ruminococcus* were the major genera in the five groups.

**Figure 6:**
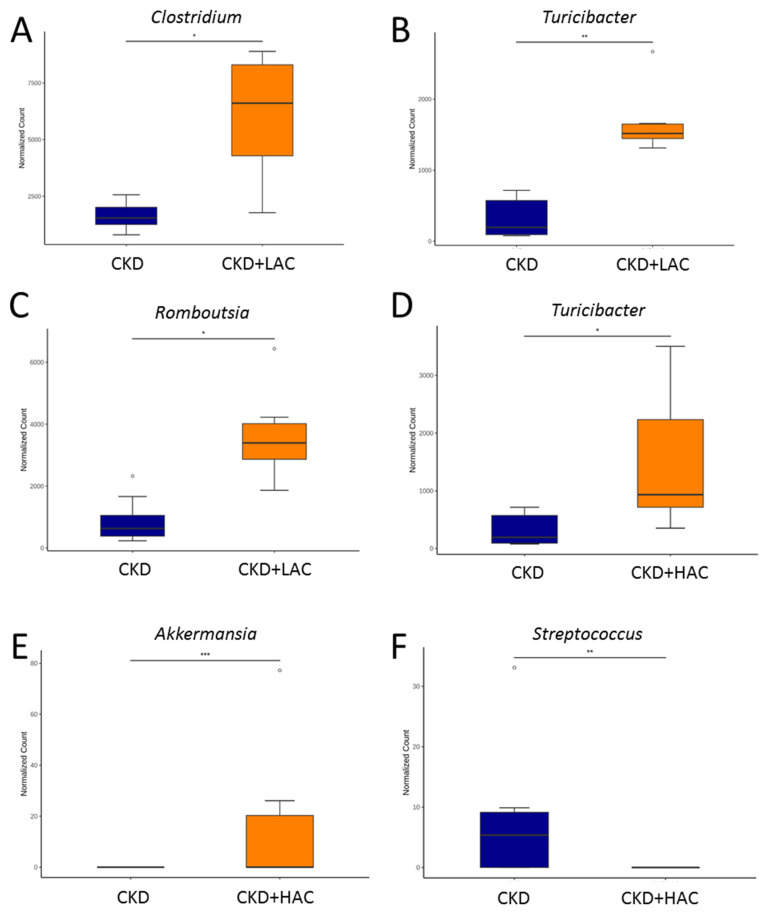
The comparison of the genera (**A**) *Clostridium*, (**B**) *Turicibacter*, and (**C**) *Romboutsia* between the CKD group and CKD + LAC group. The comparison of the genera (**D**) *Turicibacter*, (**E**) *Akkermansia*, and (**F**) *Streptococcus* between the CKD group and CKD + HAC group. N = 7–8/group. The outliers are shown as dots. * *p* < 0.05; ** *p* < 0.01; *** *p* < 0.005.

At the genus level, low-dose AC supplementation altered gut microbiota composition in CKD rats with an increase in *Clostridium*, *Turicibacter*, and *Romboutsia* (Figure 6A–C). High-dose AC treatment significantly increased the abundance of the genera *Turicibacter* (Figure 6D) and *Akkermansia* (Figure 6E), but decreased genus *Streptococcus* in the CKD + HAC group compared with the CKD group (Figure 6F).

**Figure 7:**
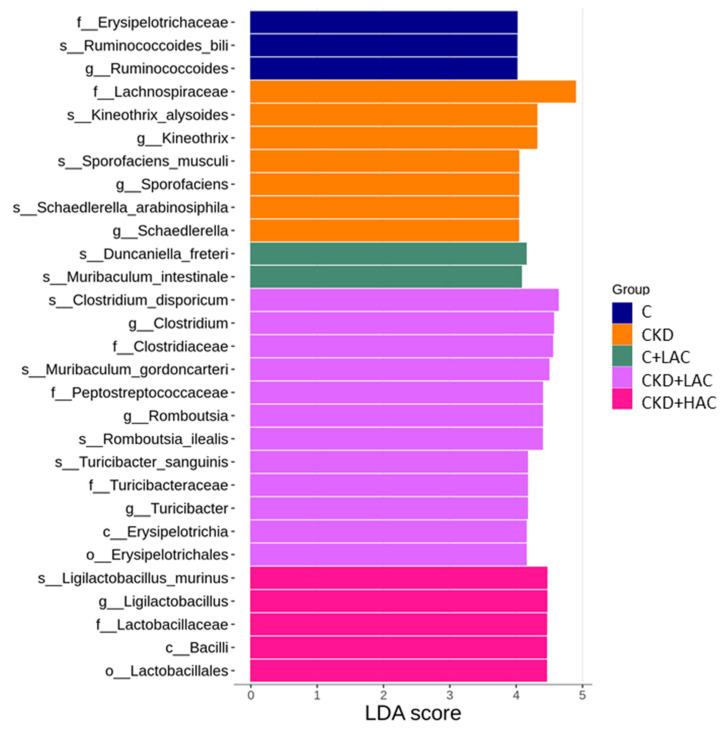
Linear discriminant analysis effect size (LEfSe) analyzed differentially abundant microbiota between five different groups. The threshold of linear discriminant analysis (LDA) score was set as 4.

The most differential abundant taxa among the five groups identified by the LEfSe analysis can be seen in Figure 7. In particular, the genera *Clostridium*, *Romboutsia*, and *Turicibacter* were more abundant in the CKD + LAC group. Additionally, high-dose AC supplementation led to a higher percentage of the species *Ligilactobacillus murinus* and the genus, family, order, and class to which it belongs.

In total, at the genus level, high-dose AC treatment exhibited a protective effect against adenine-induced CKD by enriching beneficial bacteria in the gut, such as *Akkermansia* and *Turicibacter*.

### 3.5. RAS

**Figure 8:**
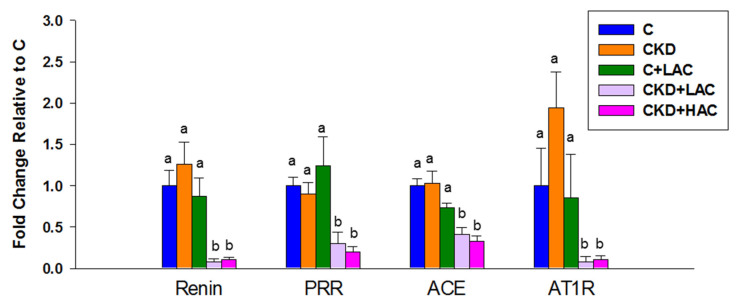
Renal gene expression of the renin-angiotensin system of rats at age 9 weeks, containing renin, (pro)renin receptor (PRR), angiotensin converting enzyme (ACE), and angiotensin II type 1 receptor (AT1R). N = 7–8/group. Statistical analysis by one-way ANOVA, *p* < 0.05. Letters represent the differences between groups.

As the RAS contributes to the regulation of BP and AC has been shown to regulate the RAS, we further evaluated whether the protective actions of AC were related to this mechanism. As observed (Figure 8), AC treatment, either low- or high-dose, caused a lower expression of renin, PRR, ACE, and AT1R in the CKD + LAC and CKD + HAC groups compared with the CKD group. However, AC treatment had a negligible effect on the RAS in the C + LAC group.

## 4. Discussion

The current research appears to be the first to show that AC treatment can improve renal outcomes in a pediatric CKD rat model. Our study provides a deeper insight into the protective function of AC in CKD through the regulation of the gut microbiota metabolites and the RAS.

Our salient findings specifically revealed as follows: (1) juvenile rats fed with 0.5% adenine developed the typical characteristics of CKD at age 9 weeks, which could be used as a model to evaluate pediatric CKD; (2) AC treatment, either low- or high-dose, similarly improved kidney function and proteinuria, and attenuated hypertension; (3) Although low-dose AC treatment increased plasma concentrations of SCFAs, affecting SCFA concentrations might be not related to the protective actions of AC against CKD; (4) CKD caused an increase in the renal expression of GPR43 and OLFR78, which was restored by AC treatment; (5) AC acts like a prebiotic by enriching beneficial bacteria in the gut, such as *Akkermansia* and *Turicibacter*; (6) the protective effect of high-dose AC might be related to enhancement of species *Ligilactobacillus murinus*; and (7) the beneficial action of AC against CKD-related hypertension might be connected to inhibition of the RAS. 

In support of the renoprotective and anti-hypertensive effects of AC [13,14], our study revealed that AC treatment protected juvenile rats against CKD and hypertension. A previous study reported the acute effect of solid-state cultured AC treatment in reducing BP in spontaneously hypertensive rats (SHRs); however, the chronic effect of AC on BP has not been fully determined in humans and animals. Our study is the first to demonstrate that low- and high-dose AC treatments have the potential to lower systolic BP by 8- and 12 mm Hg, respectively, in juvenile CKD rats after six weeks of treatment. Although the pharmacological effects of the extracts of AC in different in vitro studies show a dose-dependent manner [23], our data do not support that AC dose-dependently improved kidney function, proteinuria, and hypertension in this juvenile rat CKD model.

Although AC does not affect α-diversity, both low- and high-dose AC treatments alter β-diversity and result in distinct enterotypes. The protective effects of AC may be attributed at least in part to alterations of the gut microbiota. Our data revealed that high-dose AC increased the genera *Akkermasia* and *Turicibacter*, as well as the species *Ligilactobacillus murinus*. According to our data, low-dose AC treatment augmented the abundance of the genera *Clostridium*, *Romboutsia*, and *Turicibacter*.

Our results reconfirm prior research showing that medicinal mushrooms have prebiotic properties [12], resulting in enriching beneficial bacteria in the gut and increased production of beneficial metabolites like SCFAs. However, results from our study reveal that AC treatment has a negligible influence on another microbial metabolite, TMAO. *Akkermasia* and *Clostridium* spp. are well-known beneficial bacteria [24]. Similar to *Akkermasia* and *Clostridium*, *Turicibacter* belongs to SCFA-producing bacteria, although relatively little is known regarding its prebiotic activity. AC caused an increase in most SCFAs in plasma, but we found that CKD did not change the concentrations of SCFAs in plasma.

SCFAs regulate BP in a manner that is differentially modulated by the activities of multiple SCFA receptors [20]. The activation of GPR 43 and OLFR78 can increase BP. Conversely, they can be offset by GPR41 resulting in vasodilatation [20]. We found that CKD enhanced renal expression of GPR43 and OLFR78 and these increases were suppressed by AC treatment. As activation of GPR43 and OLFR78 was in favor of increasing BP, the anti-hypertensive effect of AC treatment would, therefore, be related to SCFA receptor signaling pathways.

Consistent with findings in hypertensive people and animals [25,26], we observed that BP was negatively correlated with the elevated abundance of the genera *Romboutsia*, *Turicibacter*, *Clostridium*, and *Akkermansia*, while positively associated with the increased abundance of *Streptococcus*. In SHRs, a low abundance of *Ligilactobacillus murinus* correlates with hypertension. Conversely, treatment with *Ligilactobacillus murinus* lowered BP in SHRs [27]. As *Ligilactobacillus* has been gaining attention as a probiotic [28], our data raise the possibility that the anti-hypertensive actions of AC may be related to its prebiotic properties to regulate hypertension-related taxa.

Another protective action of AC might involve its ability to inhibit the RAS. Aberrant activation of the RAS is an important mechanism underlying CKD progression and hypertension [29]. Our current study found that AC treatment decreased renal expression of renin, PRR, ACE, and AT1R. Considering that blockade of the RAS has a vasodilatory effect, our data agree with prior research demonstrating that AC has ACE inhibitory activity [13], by which AC treatment protected juvenile CKD rats against hypertension in this model.

The current study had a few limitations to acknowledge. Firstly, the extracts of solid-state cultures were used to evaluate the protective effects of AC. As the bioactive compounds derived from AC might be different due to various artificial cultivation techniques [4], which method is most appropriate for using AC deserves further clarification. Another limitation of the research was that the organ analysis was only focused on the kidneys. The anti-hypertensive effects of AC on CKD-associated hypertension may be attributed to other organs involved in controlling BP. Thirdly, we did not examine the other reported pharmacological effects of AC. Although some protective mechanisms (e.g., antioxidant and anti-inflammatory) might contribute to AC’s renoprotective and antihypertensive effects, we were unable to examine them all in the present study. Lastly, we mainly examined the microbial metabolites SCFAs and TMAO. Even though AC achieved beneficial effects, understanding the roles of each microbe and metabolite modulated by AC and their contribution to the prevention of CKD remains a major challenge. This needs to be studied extensively to derive a proper conclusion.

## 5. Conclusions

In conclusion, our study demonstrated that treatment using a solid-state culture of AC protected CKD rats against kidney injury and hypertension. The beneficial effects of AC are related to the enhancement of beneficial microbes, regulating SCFA receptor signaling pathways, and inhibition of the RAS. Our pre-clinical investigation provides an in-depth understanding of the impact of AC targeting gut microbiota on CKD and associated complications, which may have the potential for use as a prebiotic dietary supplement to improve global kidney health.

## Figures and Tables

**Table 1:** PCR primer sequences.

Gene	Forward	Reverse
GPR41	5 tcttcaccaccgtctatctcac 3	5 cacaagtcctgccaccctc 3
GPR43	5 ctgcctgggatcgtctgtg 3	5 cataccctcggccttctgg 3
GPR109A	5 cggtggtctactatttctcc 3	5 cccctggaatacttctgatt 3
OLFR78	5 gaggaagctcacttttggtttgg 3	5 cagcttcaatgtccttgtcacag 3
Renin	5 aacattaccagggcaactttcact 3	5 acccccttcatggtgatctg 3
PRR	5 gaggcagtgaccctcaacat 3	5 ccctcctcacacaacaaggt 3
ACE	5 caccggcaaggtctgctt 3	5 cttggcatagtttcgtgaggaa 3
AT1R	5 gctgggcaacgagtttgtct 3	5 cagtccttcagctggatcttca 3
R18S	5 gccgcggtaattccagctcca 3	5 cccgcccgctcccaagatc 3

GPR41 = G protein-couple receptor 41; GPR43 = G protein-couple receptor 43; GPR109A = G protein-couple receptor 109A; OLFR78 = olfactory receptor 78; PRR = (pro)renin receptor; ACE = angiotensin converting enzyme; AT1R = angiotensin II type 1 receptor; R18S = 18S ribosomal RNA.

**Table 2:** Weight and kidney outcome at 9 weeks of age.

Group	C	CKD	C + LAC	CKD + LAC	CKD + HAC
Mortality	0%	0%	12.5%	0%	0%
Body weight (BW) (g)	225 ± 9 ^b^	266 ± 6 ^a^	212 ± 7 ^b^	276 ± 7 ^a^	248 ± 9 ^a^
Left kidney weight (KW) (g)	1.16 ± 0.05 ^c^	1.65 ± 0.05 ^b^	1.1 ± 0.04 ^c^	1.98 ± 0.12 ^a^	1.69 ± 0.1 ^a^
Left KW/100 g BW	0.52 ± 0.01 ^b^	0.62 ± 0.01 ^a^	0.52 ± 0.01 ^b^	0.72 ± 0.05 ^a^	0.69 ± 0.04 ^a^
Creatinine (μM/L)	11 ± 10.6 ^b^	16.4 ± 1.1 ^a^	12.1 ± 0.9 ^b^	11.7 ± 0.2 ^b^	14.1 ± 0.7 ^a^
CCr (ml/min)	2.2 ± 0.2 ^a^	1.5 ± 0.2 ^b^	3.6 ± 0.5 ^a^	3.2 ± 0.2 ^a^	2.4 ± 0.3 ^a^
24 h urinary protein (mg/100 g BW)	36.4 ± 2.2 ^b^	51.3 ± 7.8 ^a^	34.3 ± 2.1 ^b^	34 ± 3.7 ^b^	41.9 ± 4.3 ^a^
Glomerular injury score	0.2 ± 0.1 ^b^	1.9 ± 0.5 ^a^	0.3 ± 0.2 ^b^	0.4 ± 0.4 ^b^	0.5 ± 0.4 ^a^
Tubulointerstitial injury score	0.2 ± 0.2 ^c^	2.5 ± 1.1 ^a^	0.2 ± 0.1 ^c^	0.6 ± 0.5 ^b^	0.8 ± 0.4 ^b^

N = 7–8/group; CCr = clearance of creatinine; statistical analysis by one-way ANOVA, *p* < 0.05. Letters represent the differences between groups.

**Table 3:** Plasma SCFA levels at nine weeks of age.

Group	C	CKD	C + LAC	CKD + LAC	CKD + HAC
Acetic acid (μM)	641 ± 51 ^b^	639 ± 39 ^b^	1240 ± 57 ^a^	636 ± 25 ^b^	734 ± 34 ^b^
Propionic acid (μM)	4.2 ± 0.3 ^b^	4.2 ± 0.2 ^b^	8.6 ± 0.5 ^a^	4.1 ± 0.1 ^b^	4.6 ± 0.3 ^b^
Isobutyric acid (μM)	1.0 ± 0.2 ^b^	0.9 ± 0.1 ^b^	1.9 ± 0.3 ^a^	0.9 ± 0.1 ^b^	1.4 ± 0.2 ^a^
Butyric acid (μM)	10.4 ± 1.1 ^b^	10.6 ± 0.6 ^b^	21.5 ± 1.2 ^a^	10.3 ± 0.3 ^b^	11.9 ± 0.6 ^b^
Isovaleric acid (μM)	22.5 ± 1.6 ^b^	22.5 ± 0.8 ^b^	46.4 ± 1.8 ^a^	21.7 ± 0.6 ^b^	23.6 ± 0.9 ^b^
Valeric acid (μM)	1.8 ± 0.4	1.9 ± 0.2	2.9 ± 0.5	1.7 ± 0.1	1.6 ± 0.4

N = 7–8/group; statistical analysis by one-way ANOVA, *p* < 0.05. Letters represent the differences between groups.

**Table 4:** Plasma concentrations of DMA, TMA, and TMAO at nine weeks of age.

Group	C	CKD	C + LAC	CKD + LAC	CKD + HAC
DMA	85 ± 7	89 ± 2	103 ± 11	94 ± 4	86 ± 3
TMA	5 ± 0.6	3.5 ± 0.2	5.3 ± 1	4.2 ± 0.5	3.4 ± 0.4
TMAO	264 ± 11	246 ± 8	269 ± 14	284 ± 24	280 ± 15

N = 7–8/group. DMA = dimethylamine; TMA = trimethylamine; TMAO = trimethylamine-N-oxide.

## Data Availability

Data are contained within the article.
